# Equity in Health Care Financing in Low- and Middle-Income Countries: A Systematic Review of Evidence from Studies Using Benefit and Financing Incidence Analyses

**DOI:** 10.1371/journal.pone.0152866

**Published:** 2016-04-11

**Authors:** Augustine Asante, Jennifer Price, Andrew Hayen, Stephen Jan, Virginia Wiseman

**Affiliations:** 1 School of Public Health and Community Medicine, University of New South Wales, Sydney, Australia; 2 The George Institute for Global Health, Sydney, NSW, 2000, Australia; 3 The University of Sydney, Sydney, NSW, 2006, Australia; 4 Department of Global Health and Development, London School of Hygiene and Tropical Medicine, London, United Kingdom; Taipei Medical University, TAIWAN

## Abstract

**Introduction:**

Health financing reforms in low- and middle- income countries (LMICs) over the past decades have focused on achieving equity in financing of health care delivery through universal health coverage. Benefit and financing incidence analyses are two analytical methods for comprehensively evaluating how well health systems perform on these objectives. This systematic review assesses progress towards equity in health care financing in LMICs through the use of BIA and FIA.

**Methods and Findings:**

Key electronic databases including Medline, Embase, Scopus, Global Health, CinAHL, EconLit and Business Source Premier were searched. We also searched the grey literature, specifically websites of leading organizations supporting health care in LMICs. Only studies using benefit incidence analysis (BIA) and/or financing incidence analysis (FIA) as explicit methodology were included. A total of 512 records were obtained from the various sources. The full texts of 87 references were assessed against the selection criteria and 24 were judged appropriate for inclusion. Twelve of the 24 studies originated from sub-Saharan Africa, nine from the Asia-Pacific region, two from Latin America and one from the Middle East. The evidence points to a pro-rich distribution of total health care benefits and progressive financing in both sub-Saharan Africa and Asia-Pacific. In the majority of cases, the distribution of benefits at the primary health care level favoured the poor while hospital level services benefit the better-off. A few Asian countries, namely Thailand, Malaysia and Sri Lanka, maintained a pro-poor distribution of health care benefits and progressive financing.

**Conclusion:**

Studies evaluated in this systematic review indicate that health care financing in LMICs benefits the rich more than the poor but the burden of financing also falls more on the rich. There is some evidence that primary health care is pro-poor suggesting a greater investment in such services and removal of barriers to care can enhance equity. The results overall suggest that there are impediments to making health care more accessible to the poor and this must be addressed if universal health coverage is to be a reality.

## Introduction

Concerns about the poor not getting adequate access to quality health care are widespread in low- and middle-income countries (LMICs). Governments, development agencies and civil society organisations have highlighted the enormous gap in access to health services in many countries and called for effective strategies to improve equity [1]. Globally an estimated 1.3 billion people do not have access to effective and affordable health care, and of those who have access about 170 million are forced to spend more than 40 percent of their household income on medical treatment [2]. Financial barriers are a key limitation to accessing health services in LMICs where out-of-pocket (OOP) payments finance a significant proportion of health expenditure. In 33 mostly low-income countries, direct OOP payments represented more than 50% of total health expenditures in 2007 [3]. There is high probability of many households in LMICs being pushed into poverty when faced with substantial medical expenses, particularly when this is combined with a loss of income due to ill-health [4]. Indeed it is increasingly recognised that measures to promote financial protection through universal health coverage (UHC) represent major components in global efforts to fight poverty and this is reflected in the UN Sustainable Development Goals [5].

UHC stipulates that people should be able to access the health services they need without risking financial ruin or impoverishment [6,7]. Effective implementation of this principle requires a robust health financing system which guarantees a fair distribution of the burden of paying for health care according to ability-to-pay (ATP) and benefits from health care spending according to need [8,9]. Health systems in many LMICs are financed through key sources such as taxation, social health insurance contributions, private health insurance premiums, and out-of-pocket payments [10]. Not all these financing sources promote equity and therefore facilitate the move towards UHC. Government spending on health, although not explicitly stated, is generally expected to benefit the poor more than the better-off [11].

Benefit incidence analysis (BIA) and financing incidence analysis (FIA—also referred to as progressivity analysis) are the most well recognised tools for assessing the extent to which public spending on health benefit the poor and are increasingly being used by governments and international agencies such as the World Bank to assess progress toward UHC targets [12,14]. BIA estimates the distributional impact of public spending on health care. It measures the extent to which different socio-economic groups benefit from public subsidies through their use of health services [8,13]. A key step in the computational process is to subtract any out-of-pocket payment made in the course of using health services in order to arrive at the actual subsidy received [15] (Box 1). FIA assesses the distribution of the burden of health financing across socio-economic groups, and the extent to which this burden affects the underlying distribution of income [16].

**Box 1. Measuring benefit and financing incidence.**

**Table:** 

Approach	Description
BIA	Undertaking BIA involves following a number of steps: ranking the study population by a living standard measure, assessing the rate of utilisation of different types of health services, estimating the unit cost of each type of service, and multiplying the utilisation rates and unit costs to determine the amount of subsidy. Direct user fees are deducted before arriving at the final amount of government subsidy [15]. The amount of subsidy is usually the difference between the costs incurred for providing the services and the fees paid by the user expressed as:
	*S*_*ki =*_ *C*_*ki*_*−F*_*ki*_ *= c*_*ki*_*−f*_*ki*_*q*_*ki*_ *= q*_*ki*_ *(c*_*ki*_*−f*_*ki*_*) = s*_*ki*_*q*_*ki*_
	where *S*_*ki*_ are the subsidies that individual *i* receives from subsector *k* (e.g. hospital inpatient care), *C*_*ki*_ are the costs incurred by providers in subsector *k* in providing services to individual *i*, *F*_*ki*_ are the fees paid by individual *i* to the provider in subsector *k*, *q*_*ki*_ is the number of units of service of type *k* consumed by individual *i*, and *c*_*ki*_, *f*_*ki*_ and *s*_*ki*_ are the unit costs, fees and subsidies, respectively, for sector *k* for individual *i*. At the individual level, only *F*_*ki*_ and *q*_*ki*_ are recorded in the household survey data. The goal of BIA is to estimate the distribution of the *S*_*ki*_ by income [13].
FIA	A key indicator for measuring the progressivity of a health financing system is the Kakwani index (KI) [63], which is defined as twice the area between the concentration curve of health payments and the Lorenz curve [17]. The KI is calculated as:
	*πK = C–G*
	where *C* is the concentration index for health payments and *G* is the Gini coefficient of the ATP variable. The value of *πK* ranges from -2 to 1. A negative value indicates a pro-rich or regressive health care payment system. A positive value indicates a progressive financing system with the concentration curve of health care payment lying outside the Lorenz curve. Where health care payment is proportional to ATP, the concentration curve lies on top of the Lorenz curve and the index is zero [12].

To maintain an equitable health financing system, it is generally accepted that payment for health care must to be on the basis of ATP. FIA or progressivity analysis measures the departure from proportionality in the relationship between payments for health care and ATP [8,17]. The progressivity of a health care financing system is computed in two phases–first, by computing the progressivity of each source of health care finance, and second, by weighting the progressivity of the different financing sources by their shares in total health finance often estimated from National Health Account data to obtain overall progressivity. A progressive financing system is one in which households with higher income contribute a higher share of their income towards health than do those with lower income and a regressive system is the opposite [6,10].

While several countries have reformed their health care sector over the last decades to move towards a more pro-poor system, the evidence on progress towards equitable health financing in LMICs overall remains sketchy. A recent systematic review by Anselmi and colleagues [18] focuses on equity in allocation of public health sector expenditures in LMICs. It does not cover the distribution of the burden of financing the health system across households with different ATP–a key aspect of health financing equity. This systematic review builds on Anselmi’s work by assessing the evidence on health financing equity in LMICs drawing specifically on studies that use BIA and FIA as the principal methodology and were published between 1994 and October 2013. The aim is to provide evidence on advancement in health financing equity in LMICs to inform the debate on universal health coverage. The 20-year period chosen was to enable the capture of more recent evidence. Insights from this review may be useful for assessing the performance of health systems in LMICs and also help policymakers and planners to further strengthen their current health financing arrangements.

## Methods

### Ethics

This is a systematic review and requires no ethics approval. However, the entire study under which this review is conducted has been approved by the Human Research Ethics Committee of UNSW Australia (Approval number: HC13269); the Fiji National Health Research Committee (Approval # 201371); and the Timor-Leste Ministry of Health (Ref MS/UNSW/VI/218).

### Search strategy

We searched the published and unpublished literature including the following bibliographic databases: Medline, Embase, Scopus (Health Sciences and Social Sciences and Humanities), Global Health and CinAHL. We also searched key Economics databases including EconLit and Business Source Premier. Bibliographies of identified articles and reports on BIA and FIA were examined to obtain additional documents. Finally, key individuals with expertise in BIA and FIA were contacted for personal recommendations. For the grey literature we searched the websites for relevant organisations supporting health care in LMICs including World Bank, United Kingdom Department for International Development (DFID), Asian Development Bank (ADB), World Health Organization (WHO), United States Agency for International Development (USAID) and the Organisation for Economic Co-operation and Development (OECD).

A range of key terms related to equity in health financing generally, and more specifically, benefit and financing incidence analyses, were used for the electronic database searches. They included: (equity OR inequity OR equitable OR inequitable OR pro-poor OR pro-rich OR progressiv* OR regressiv* OR proportio*) AND (public OR government OR state) AND (health OR health care OR health system financ* OR paymen* OR expenditur* OR spending OR subsid* OR subsidized) AND (benefit incidence OR benefit incidence analysis OR bia OR financing incidence OR financing incidence analysis) AND (low and middle income OR lmic* OR developing OR least developed countr*). Different combination of these search terms were applied to the various databases and the search was limited to 1994 and October 2013 and also to low- and middle income- countries. Low-income countries, according to the World Bank, are those with a GNI per capita of $1,045 or less in 2013, while middle-income countries are those with a gross national income (GNI) per capita of more than $1,045 but less than $12,746 [19]. All the countries included were classified as low and middle income at the time the review was undertaken. The electronic search was undertaken independently by two researchers (AA and JP).

### Screening and selection of studies

The selection of studies to be included in this review was done in three stages based on pre-developed selection criteria (Table 1). At stage 1, we checked all the search results (n = 512) to remove duplicates, non-English references, and references that were outside the stipulated time frame. Three potentially useful non-English references were excluded. The screening was done independently by two reviewers (AA and JP). The stage 2 screening involved the two independent reviewers reading titles and abstracts of all references that passed the stage 1 screening (n = 305) to assess their relevance to the topic. In the final stage, the full texts of the references that passed the stage 2 screening (n = 87) were assessed against the selection criteria for the final selection of studies to be included in the review (n = 24). Differences in opinion at any stage of the screening process were discussed and resolved before moving to the next stage. Single papers that apply both BIA and FIA in one country were treated as one study with the results reported under the appropriate headings. However, the results of papers that focus on multiple countries were disaggregated by country and then by method of analysis (BIA or FIA). For the FIA, we excluded studies that focus on only one health financing mechanism such as OOP spending or social health insurance because determining the progressivity of a health financing system requires an assessment of all financing sources in the country [6,20]. We built an Endnote library for all potentially relevant studies that passed the stage 1 screening.

**Table 1:** Inclusion and exclusion criteria.

Criteria	Inclusion	Exclusion
Time period	From January 1994 to October 2013	Before 1994
Language	English	Non-English
Origin of study	Sub-Saharan Africa, Asia-Pacific, Latin America, Middle East	Developed countries
Methodology	BIA & FIA (including progressivity analysis)	Health financing but not using BIA or FIA
Dimension of study	Single & multi-country	Studies focusing on concept or methodology
	FIA studies reporting on all financing mechanisms	Studies focusing on only one financing source (e.g., out-of-pocket) or only one service (e.g. anti-retroviral therapy or ART financing)
	Studies focusing exclusively on health sector	Studies comparing health with other sectors

### Data extraction

Two researchers (AA and JP) critically reviewed all the papers included in the study for evidence on equity in health financing. Each paper was reviewed independently and information was extracted into a database using a pre-established standard data entry format. We followed the Preferred Reporting Items for Systematic Reviews and Meta-Analyses (PRISMA) checklist in gathering the relevant information (S1 PRISMA Checklist). Basic information describing each of the studies such as names of authors, title, year of publication, country of focus, method and data sources, key findings, and main conclusions were extracted. Any differences between the two reviewers in terms of data extracted were resolved by consensus among all authors. Overall, less than 5% of data extracted were discrepant and were resolved by consensus. Appraisal of the quality of each study during the review and data extraction phase was carried out paying particular attention to methodological soundness, the appropriateness of the study design and the overall weight of evidence on health financing equity.

The data extracted were largely qualitative although the concentration and Kakwani indices were reported for the majority of the BIA and FIA studies. The BIA studies typically reported on whether the distribution of public subsidy across different socioeconomic groups was pro-poor or pro-rich at various levels of a health system (e.g. primary, secondary and/or tertiary), service types (e.g. outpatient/inpatient) and/or sector (public/private) depending on data availability. The FIA studies reported on whether, and the degree to which, payment for health care has been progressive, regressive or proportional across households as ranked by their ATP. Each member of the review team critically examined the evidence extracted into the database table to ensure it addressed the objective of the review.

We assessed the risk of bias in each study at both the study and outcome levels. For all the studies we critically reviewed the methodology, including whether approaches followed were sufficiently described, sources of data adequately reported, and findings appropriately presented. We have published a protocol that outlines the key steps for conducting BIA and FIA and the type of data required: http://bmjopen.bmj.com/content/4/12/e006806.full. We assessed the extent to which each study followed established methodologies for computing the benefits or burden from health care. For the BIA studies, the assessment of risk of bias included whether nationally representative household datasets were used or whether primary data was collected; and if so, whether there was any sampling bias. We further assessed the extent to which the utilisation data allowed for reasonable calculation of rate of health service use and how unit cost was calculated. For the FIA studies, we followed the recommended methodological approach in key studies including O’Donnell et al. [12]; Mills et al. [6]; Akazili et al. [21] and Limwattananon et al. [9] by assessing whether the progressivity of each health financing source was first computed before aggregating to obtain the progressivity of the overall health financing system. We also assessed whether the FIA studies reported concentration and Kakwani indices, and whether sensitivity analysis was performed. Studies that had gaps based on their assessment, were considered to have weak evidence on health financing equity.

### Analysis of data

The BIA studies included in this review (n = 18) differ in terms of the way results are presented. We classified the studies into three groups: those presenting concentration indices (CIs) to illustrate the extent of equity in the distribution of health care benefits (n = 12); those presenting percentage shares of subsidy received by population quintiles or deciles (n = 5); and those using only concentration curve (CC) to depict equity in the distribution of benefits (n = 1). Some of the studies combined different approaches, especially percentage share of subsidy and concentration index.

To analyse the data extracted from the BIA studies in a uniform manner, we developed a scale (from -3 to 3) to score each study based on the degree of equity in distribution of benefits reported. For those studies presenting CIs illustrating pro-poor distributions, we assigned a value of 1 where CI < 0.1; 2 where CI = >0.1< = 0.2; and 3 where CI > 0.2. The choice of midpoints was informed by the range of concentration indices reported across the studies. Negative values were assigned where the distribution is pro-rich and a score of zero is assigned where it is proportional. It must be noted that the values assigned to score the studies are independent of whether the reported concentration index is negative or positive. For studies presenting percentage share of subsidy by quintiles or deciles and without CIs, we applied the same scale but using different thresholds: 1 where the poorest quintile/decile received less than 5% share of subsidy over and above its population share; 2 where 5–10% is received; and 3 where more than 10% is received. We assigned negative values using the same scale where the distribution is pro-rich and a zero where it is proportional. Finally, for those studies using concentration curves to illustrate the extent of equity, we applied the same scale (-3 to 3) but the thresholds were based on observation of the CC and its proximity to the 45 degree equality line (see Box 2 for summary of approach).

**Box 2. Summary of approach to scoring the BIA studies.**

**Table:** 

a) Studies reporting concentration index	where
	CI <0.1 assign 1 if pro-poor or -1 if pro-rich
	CI = > 0.1 < = 0.2 assign 2 if pro-poor or -2 if pro-rich
	CI > 0.2 assign 3 if pro-poor -3 if pro-rich
b) Studies reporting % shares of subsidy by population quintile or decile	where
	share of poorest or richest group is <5% above population share assign 1 if pro-poor or -1 if pro-rich
	share of poorest or richest group is = >5% < = 10% above the population share assign 2 if pro-poor or -2 if pro-rich.
	share of the poorest or richest group is > 10% above population share assign 3 if pro-poor or -3 if pro-rich
c) Studies using concentration curve to depict equity of distribution	Assign score -3 to 3 depending on the proximity of the concentration curve to the 45 degree line of equality.

The FIA studies were more uniform in their approach to presenting findings: combining concentration with Kakwani indices to measure the degree of progressivity. We analysed the FIA data by comparing the Kakwani indices for the different sources of financing and their corresponding total indices. Because of the qualitative nature of the data from the BIA and FIA studies coupled with differences in the study settings, meta-analysis could not be undertaken; instead, a narrative synthesis approach [22] was employed in the analysis and presentation of the overall evidence. The lead reviewer (AA) drafted the overall report which was checked and commented on independently by all authors.

## Results

### Selection process and outcome

**Fig 1:**
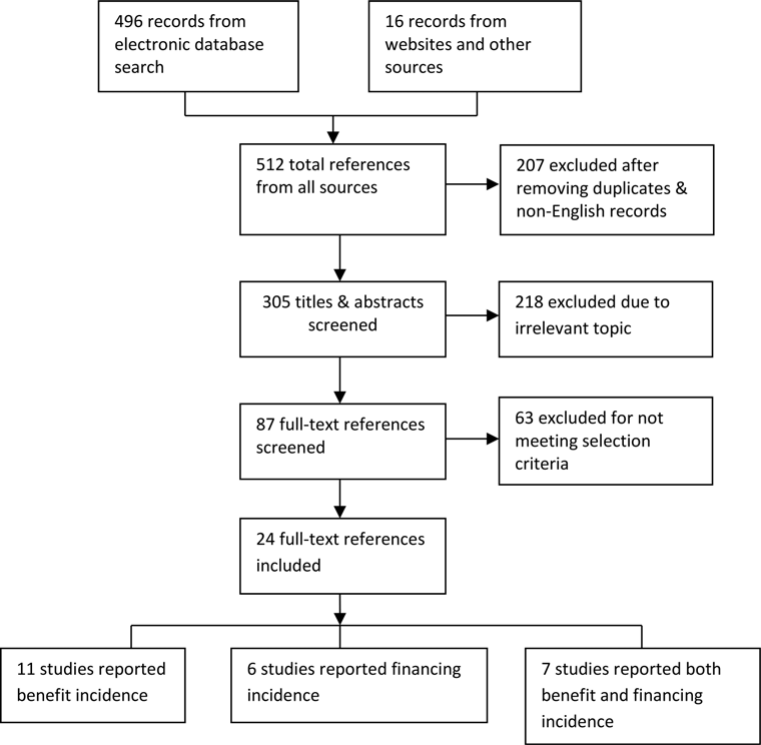
PRISMA Flow Diagram for Selection of Studies.

A total of 512 references were retrieved from the electronic databases and websites of development agencies. The full texts of 87 references were assessed against the selection criteria and 24 studies were judged appropriate for inclusion. Of the 24 studies, 11 reported exclusively on BIA, six on FIA and seven on both BIA and FIA (Fig 1).

### Study characteristics

A total of 18 BIA studies compared to 13 FIA were obtained from the 24 papers after separating those papers that combined the two methodologies (Table 2). Fifty percent (n = 12) of the papers included in the review originated from sub-Saharan Africa, 38% (n = 9) from the Asia-Pacific region and 12% (n = 3) from Latin America and Middle East combined. About 71% (n = 17) of the papers were published between January 2011 and October 2013, indicating recent growth in the popularity of BIA and FIA for measuring health care financing equity in LMICs. In terms of country of focus, although more studies originated from sub-Saharan Africa than from the Asia-Pacific region, there were more countries covered in Asia-Pacific (13) than in sub-Saharan Africa (10) and this is primarily because of two large multi-country studies undertaken in Asia-Pacific in 2007 and 2008 [11,12]. Some countries in these regions including Kenya, Ghana, and Thailand had multiple BIA and/or FIA studies. The World Bank published seven of the 24 papers–four in sub-Saharan Africa and three in Asia-Pacific. The papers originating from China, India and Nigeria were partial analysis focusing on selected provinces or states rather than the entire country [11,12,23,24,25]. All the studies used either existing secondary datasets from nationally or regionally representative surveys or a combination of secondary and primary data. Four papers from sub-Saharan Africa gathered primary data to complement the existing secondary household survey and other datasets [6,21,26,27]. These four papers also focused on both the public and private sectors. From a methodological or reporting perspective, no risk of bias was identified within or across the studies included in this review as they all followed standard approaches for undertaking and reporting BIA and FIA results. However, potential risk of sampling bias could not be ruled out for the BIA studies which gathered primary data from sampled districts.

**Table 2:** Characteristics of studies included in the review.

Author	Year	Country	Methodology		Data sources		Level of analysis		Sector	
			BIA	FIA	Primary	Secondary	National	Sub-national	Public	Private
**Sub-Saharan Africa**										
Onwejekwe et al	2012	Nigeria	X		X			X	X	
Chuma et al.	2011	Kenya	X		X	X	X		X	X
Munge and Briggs	2013	Kenya		X		X	X		X	
Mills et al.	2012	Ghana, Tanzania & South Africa	X	X	X	X	X		X	X
Akazili et al.	2012	Ghana	X	X	X	X	X		X	X
Mangham	2006	Malawi	X			X	X		X	
Mtei et al.	2012	Tanzania	X	X	X	X	X		X	X
Castro-Leah et al.	2000	Cote d’Ivoire, Ghana, Guinea, Kenya, Madagascar, South Africa and Tanzania	X			X	X		X	
World Bank	2012	Ghana	X	X		X	X		X	
World Bank	2012	Kenya	X			X	X		X	
World Bank[59]	2012	Malawi	X			X	X		X	
World Bank	2012	Zambia		X		X	X		X	
**Asia-Pacific**										
Limwattananon et al.	2011	Thailand	X	X		X	X		X	
Yu et al.	2008	Malaysia		X		X	X		X	
Chen et al.	2012	China (Gansu Province)		X		X		X	X	
Chakraborty et al.	2012	India	X			X		X	X	
World Bank[60]	2012	Mongolia	X	X		X	X		X	
World Bank [61]	2012	Pakistan	X			X	X		X	
World Bank [62]	2012	Vietnam	X	X		X	X		X	
O’Donnell et al.	2007	Japan*, Hong Kong*, Korea*, Taiwan*, Indonesia, China, Thailand, Bangladesh, Sri Lanka, Punjab (India) Kyrgyz, Nepal, Philippines.	X			X	X	X	X	
O'Donnell et al.	2008	Japan*, Hong Kong*, Korea*, Taiwan*, Indonesia, China, Thailand, Bangladesh, Sri Lanka, Punjab (India) Kyrgyz, Nepal, Philippines.		X		X	X	X	X	
**Other LMICs**										
Uga and Santos	2007	Brazil		X		X	X		X	
Angeles et al.	2007	Ecuador	X			X	X		X	
Halasa & Nassar	2010	Jordan	X			X	X		X	

* Not included in the analysis as they are not classified as low and middle income.

### Distribution of health financing benefits and burden

#### Sub-Saharan Africa

**Fig 2:**
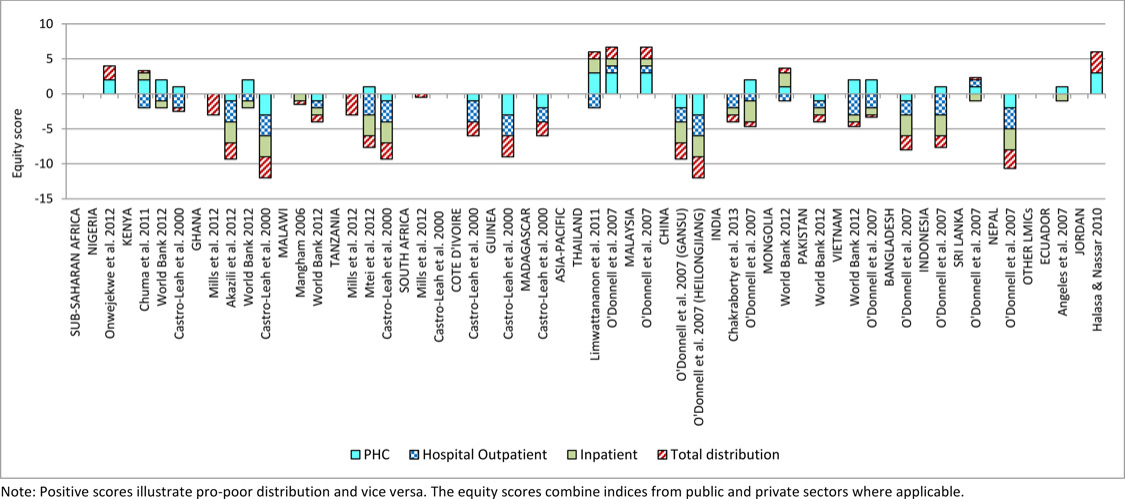
Equity scores from the BIA studies depicting the distribution of health care benefits.

Evidence from the BIA studies demonstrates consistently pro-rich distributions of total health financing benefits in sub-Saharan Africa. The better-off in most of the countries benefited more from health care spending through their use of health services than the poor (Fig 2). About 78% (n = 14) of all the BIA studies from sub-Saharan Africa reported a pro-rich distribution of health care benefits (Fig 2). At the hospital outpatient level, the pro-rich distribution was particularly strong with 12 out of 14 studies (86%) reporting this finding compared to 7 out of 15 (47%) at the primary health care (PHC) level. Ten of the BIA studies from sub-Saharan Africa reported the distribution of inpatient care and 80% found it pro-rich. About 40% (n = 6) of the studies from sub-Saharan Africa reported pro-poor distribution of health care benefits at the PHC level (Fig 2).

In terms of individual countries, only the BIA study from Nigeria [25] focusing on a limited set of priority public health services found the total distribution of benefits to be pro-poor with a concentration index of -0.11. However, this finding was not surprising as the study focused on a set of priority health services freely provided at PHC levels and no hospital level distribution was reported (Fig 2). For the majority of the countries in sub-Saharan Africa, the distribution of total benefits was pro-rich despite the distribution of PHC services favouring the poor in some countries. In Ghana for example, the World Bank [28] found that the distribution of PHC benefits to be pro-poor but the total distribution, under different unit cost assumptions, was pro-rich largely as a result of hospital level care that benefited the better-off. Chuma et al. [26] obtained similar results in Kenya in 2003 where services in public PHC facilities had a pro-poor distribution but a pro-rich distribution in public and private hospitals influenced the overall distribution to become pro-rich. In Malawi and South Africa the total distribution of benefits was close to proportional as evident in the BIA studies by Mangham [29], Mills et al. [6] and Castro-Leal et al [30].

Several of the BIA studies from sub-Saharan Africa covered both the public and private sectors in a system-wide analysis. The study by Chuma et al. in Kenya [28], Mills et al. in Ghana, Tanzania and South Africa [6]; Akazili et al. in Ghana [21] and Mtei et al. in Tanzania [27] all adopted a system-wide approach looking at both the public and the private sector. Apart from Chuma et al. the rest of these studies were part of a larger consortium to develop strategies for health insurance and equity in less developed countries (SHIELD) [21] and hence, the methodologies were similar. The World Bank study in Ghana [28] also covered the private sector.

**Fig 3:**
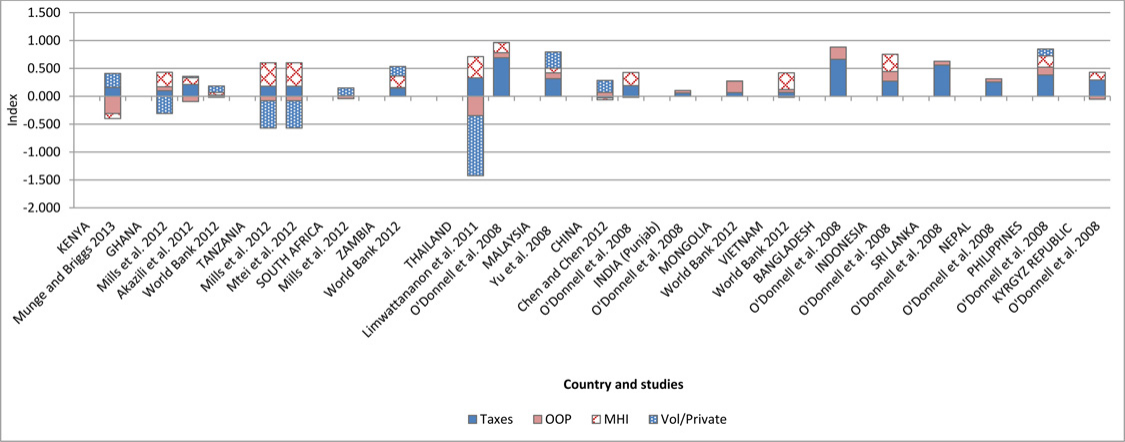
Kakwani indices for the four main financing sources reported by FIA studies from sub-Saharan Africa and Asia-Pacific.

The distribution of health financing burden in sub-Saharan Africa, based on the FIA studies evaluated, was generally progressive (Fig 3). Thus, households with higher income contributed a higher share of their income towards health care than those with lower income. About 75% (n = 6) of all the FIA studies from sub-Saharan Africa reported a progressive distribution of total health financing. The four main sources of health financing that emerged from the analysis are taxation (direct and indirect), out-of-pocket, mandatory health insurance (social health insurance), and voluntary or private health insurance. Two of these financing sources, taxation and mandatory health insurance were found by the vast majority of the FIA studies (100% for taxation and 86% for mandatory insurance) to have a progressive distribution. Indirect taxes, particularly Value Added Tax (VAT), were regressive in the majority of the FIA studies but the degree of regressivity was often not enough to overturn the highly progressive direct taxes. Out-of-pocket payments and voluntary/private health insurance were found to be regressive. OOP payment was regressive in all the countries except in Zambia where the World Bank [31] found the distribution to be proportional (7 out of 8 FIA studies from sub-Saharan Africa reported the regressivity of OOP payment). Voluntary or private health insurance was moderately regressive compared to OOP payment (50% of FIA studies found this financing source regressive).

**Fig 4:**
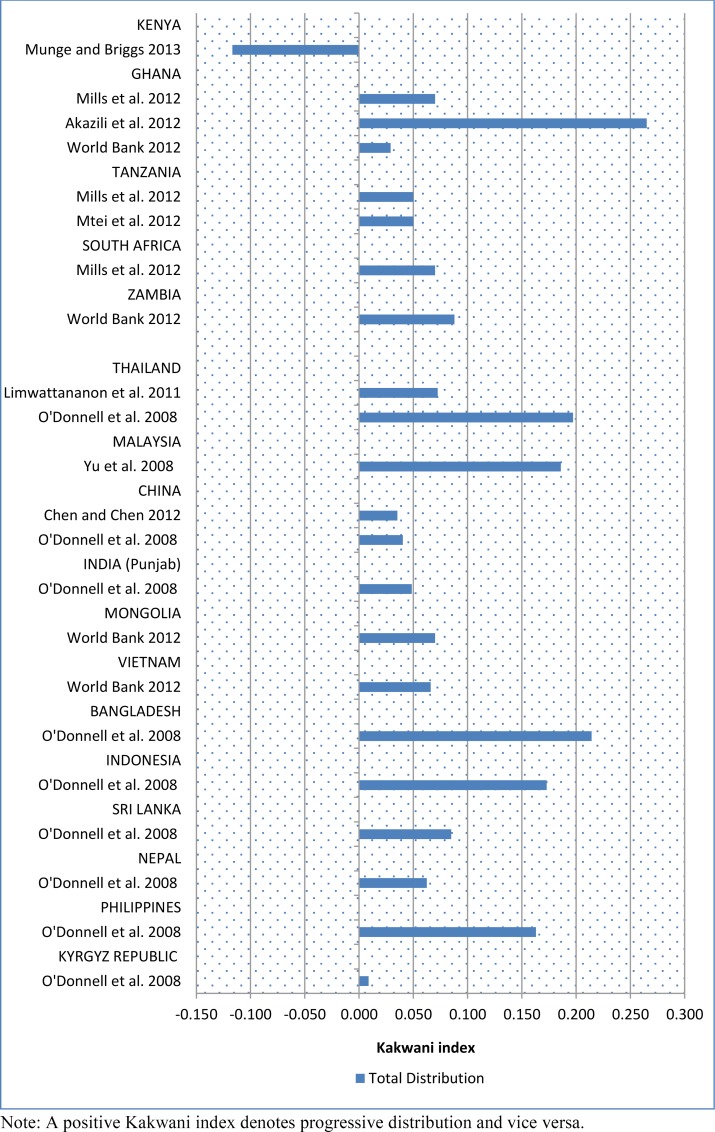
Kakwani index of total distribution of health care payments reported by FIA studies from sub-Saharan Africa and Asia-Pacific.

Based on the FIA studies from sub-Saharan Africa, Kenya was the only country where the overall distributions of the health financing burden were found to be in favour of the better-off (Fig 4). In summary, the results from the BIA and FIA studies evaluated for sub-Saharan Africa were highly consistent in pointing to a pro-rich distribution of health care benefits and a generally progressive health financing system but with significantly regressive OOP payment and mildly regressive voluntary/private insurance mechanisms.

#### Asia-Pacific

The results of studies originating from Asia-Pacific differ to some extent from those coming from sub-Saharan Africa although there were some similarities especially in regards to the BIA. About 64% (9 out of 14) of the BIA studies reported a pro-poor distribution of health care benefits at the PHC level compared to the 40% for sub-Saharan Africa (Fig 2). At the hospital outpatient level, the distribution of benefits was pro-rich similar to sub-Saharan Africa with 80% (n = 12) of studies reporting such finding, close to the 86% of studies found in sub-Saharan Africa. The distribution of inpatient care in Asia-Pacific was slightly less pro-rich than hospital outpatient care, with 73% of studies compared to 80% reporting these findings. The overall distribution of health care benefits in Asia-Pacific was pro-rich (67%) although to a lesser extent compared to sub-Saharan Africa (72%).

In terms of individual countries in Asia-Pacific, the majority of the BIA studies (60%) including those from India, Indonesia and Vietnam reported a pro-poor distribution of benefits at the PHC level and a pro-rich distribution at the hospital level (Fig 2). However, the BIA studies from China, Pakistan, Bangladesh and Nepal found a pro-rich distribution of benefits at both the PHC and hospital levels, making the distribution of total health financing benefits in these countries firmly pro-rich (Fig 2). The final set of BIA studies from Thailand, Malaysia, Sri Lanka and Mongolia found a pro-poor distribution of benefits at both the PHC and hospital levels although inpatient care in Sri Lanka and hospital outpatient in Mongolia were marginally pro-rich. These four countries appear to be making significant strides towards health financing equity. Thailand and Malaysia, in particular, seem to have established a strongly pro-poor distribution of health care benefits at all levels of the health system [9,11]. In terms of covering the private sector, only one of the BIA studies from Thailand [9] incorporated some analysis of the use of private health facilities.

The distribution of health financing burden in Asia-Pacific appears much more equitable than in sub-Saharan Africa; nearly 93% (n = 13) of all FIA studies from Asia-Pacific reported a progressive distribution of total health financing burden (Fig 3). Taxation and mandatory health insurance (social health insurance) were the most progressive financing sources with 93% and 90% respectively of the FIA studies reporting this finding. Voluntary health insurance was the least progressive with 57% (n = 4) of the studies observing this pattern of distribution. Studies from eight countries (Malaysia, Mongolia, Bangladesh, Vietnam, Indonesia, Sri-Lanka, Nepal and Philippines) reported a progressive distribution of financing burden across all financing in those countries. In several of the countries, however, although the overall distributions were progressive, some of the financing sources had a regressive distribution. An example is Thailand where Limwattananon et al. [9] reported regressive OOP payments and private health insurance, but the overall distribution was progressive and this was driven by a highly progressive tax distribution. There were similar findings from India, Kyrgyz Republic and China where at least one financing source was found to be regressive or proportional. Regarding the total distribution, China was the only country where the overall distribution of health financing burden was found to be regressive [23] (Fig 3).

One major difference between the FIA results from Asia-Pacific and sub-Saharan Africa is that the burden of OOP payment in Asia-Pacific was borne largely by the better-off while in sub-Saharan Africa the poor suffer disproportionately from direct payment. In addition to this, the better-off in Asia-Pacific also bear the largest share of the burden of paying for mandatory health insurance (largely social health insurance—SHI); all but one study reported a progressive SHI distribution. A similar result of a progressive SHI was obtained in sub-Saharan Africa. This may be due to the fact that in most countries SHI is linked to formal sector employment and as a result the burden of payment for this insurance rests largely on formal sector employees who may be relatively better-off. The distribution of voluntary/private health insurance burden in Asia-Pacific was found to be progressive albeit less so than other financing sources (Fig 2). Nonetheless, voluntary/private health insurance in Asia-Pacific appears more equitable than in sub-Saharan Africa, where it was found to be mildly regressive.

#### Other LMICs

Only three BIA and FIA studies included in this review originated outside sub-Saharan Africa and Asia-Pacific. Two of these studies are from Latin America (Brazil and Ecuador) and consist of one BIA and one FIA. The Brazilian study [32] reported on the progressivity of the health financing system and found the overall distribution to be regressive despite a progressive distribution of mandatory health insurance and voluntary/private health insurance. The regressive distribution appears to be driven largely by highly regressive OOP payments–which are similar to the picture from sub-Saharan Africa. The BIA study from Ecuador [33] found the distribution of health care benefits at the PHC level to be pro-poor but inpatient care was pro-rich making the total distribution proportional (Fig 2). The only study from the Middle-East [34] reported on the distribution of basic health care (at the PHC level) and found a strongly pro-poor pattern with the poorest group receiving 33.8% of the total benefit (equity score of 3) compared to the richest group receiving just 4% (Fig 2).

## Discussion

This systematic review assessed the evidence on health financing equity in LMICs to inform the debate about universal health coverage. The review draws exclusively on studies that use benefit and financing incidence analyses as analytical methods to evaluate health financing equity. Our findings, although have been presented separately, should be seen as complementary in terms of understanding the overall progress towards health financing equity. The results of this review can be summarised as follows: 1) the distribution of total health financing benefits in sub-Saharan Africa and Asia-Pacific is pro-rich but more so in sub-Saharan Africa than in Asia-Pacific, and this is driven by a highly pro-rich distribution of hospital services; 2) the distribution of health financing benefits at the PHC level is significantly pro-poor in Asia-Pacific but marginally so in sub-Saharan Africa; 3) the distribution of the burden of paying for health care is largely progressive in sub-Saharan Africa and Asia-Pacific with the better-off contributing a higher share of their income towards health, but variations exist in the degree of progressivity with different financing sources. Each of these findings is discussed below. The discussion focuses largely on the finding from sub-Saharan Africa and Asia-Pacific, as only three studies originated from LMICs in other regions.

The pro-rich distribution of health benefits in sub-Saharan Africa and much of Asia-Pacific warrants further attention by policy makers and researchers. This finding is not wholly surprising as it is a common feature of health systems in LMICs that the poor and rural populations experience barriers to hospital treatment [35]. It indicates that the wave of health financing reforms in these countries to promote universal coverage has some way to go to achieve their goals [36,37]. Although in principle many LMIC governments have prioritised primary health care, in practice they have not been able to shift resources in favour of the primary sector [35]. Large shares of government health spending in LMICs are still concentrated on inpatient hospital services, particularly tertiary and specialised care, most of which are urban-based and often too costly to be accessed by the poor [38]. It is crucial that timely access to secondary and tertiary services is given to all those in need of care particularly the poor.

The marginally pro-poor distribution of health benefits at the PHC level in sub-Saharan Africa, and to some extent Asia-Pacific, suggests that utilisation of PHC services by the poor, especially in sub-Saharan Africa, has not seen much improvement despite the centrality of PHC in the expansion of health services in LMICs [39]. Although traditionally health service utilisation has been lowest among the least well off [40], there has been a perception that poorer households may be using PHC services more because of their relative availability in rural areas compared to hospital services. The limited evidence of pro-poor distribution at the PHC level in this review suggests that the decade-long health reform to strengthen PHC has had only minimal impact on service utilisation by the poor. This is partly because PHC is often not well matched to the needs to the poor and therefore they may instead access private services, go directly to hospital or not seek any care at all. PHC needs to be better designed to meet the needs of the poor.

It is well recognised that affordable, effective and equitable health care requires a well-supported and comprehensive primary health care system [41]. To some extent, government investment in PHC in sub-Saharan Africa and many LMICs still lags behind that of hospital-based care [30]. There is the need to reallocate government resources towards PHC not only to enhance equity but also to improve efficiency of health systems in LMICs. Traditionally the poor tend to be the least healthy and probably have the most to benefit from health care. Therefore the greatest health gains could be realised by concentrating marginal resources on treatment of the poor [40]. Efforts to make health systems more pro-poor also need to recognise the fact that many poor people do not use health services at all, not even primary services, for a variety of reasons including quality [30]. Making health services available without addressing the factors that undermine effective use of those services will not deliver sufficient benefits to the poor.

The equity of a health financing system does not only depend on how pro-poor the distribution of its benefits is but also how the financing burden is shared. With about 93% of FIA studies from Asia-Pacific indicating that the distribution of total health financing burden is progressive, the region appears to have taken great strides towards equity in payment for health care. However, this progressive distribution, especially for OOP payment, seems inconsistent with existing knowledge that poorer households in Asia-Pacific suffer disproportionately from direct payment for health care. Out-of-pocket payment constitutes a significant portion of health financing in Asia-Pacific. In 2008, the proportion of OOP as a share of total health expenditure was greater than 50% in Bangladesh, Cambodia, India, Lao People’s Democratic Republic, Myanmar, the Philippines and Vietnam [42]. There are potential efficiency gains to be derived from the implementation of an OOP policy in LMICs [43]. Besides generating revenue to supplement the often meagre government health budget, OOP payments can enhance access to health care for the poor, especially if richer population groups pay and the revenue generated is invested in expanding access to services. It can also improve health systems’ efficiency by encouraging rational use of services and reducing inappropriate referrals’ [43]. However, as studies have demonstrated, OOP payments have a negative impact on households, especially poorer ones [44,45]. It has been observed that millions of people in Asia-Pacific, particularly the poor, are unable to use the health services they need partly as a result of OOP payment [42,44]. This suggests that although the rich may be bearing the largest burden of OOP payment in those Asian countries included in this review, the poor continue to suffer from the effects of this payment no matter how small their contributions.

The impact of OOP payment on poorer households in Africa is well documented [46,47,48] and is supported by the findings of this review which indicated that OOP payment across the continent is regressive (88% of the FIA studies found OOP payment to be regressive). Informal insurance mechanisms that households use to cope with OOP payments such as charity and use of informal credit networks are available across Africa [49]. While these mechanisms are important in abating some of the burden associated with health care costs, they do not address the source of such costs. Furthermore the policy implications of these informal coping mechanisms are unclear since they vary from one setting to another. Within the past couple of decades health reform in several African countries including Uganda and Ghana have led to the removal of user fees and a move to establish formal mechanisms such as social and voluntary health insurance schemes [46,50]. However, some of the voluntary health insurance schemes established in place of OOP payment have been found to be themselves regressive. In this review approximately 50% of the FIA studies from sub-Saharan Africa found the distribution of voluntary/private health insurance to be regressive. Some have attributed this partly to design issues, including the flat rate of premium charged to all subscribers regardless of their socioeconomic status [6]. What remains unclear however is how a voluntary payment scheme can be regressive. A recent willingness to pay study in Malaysia found a higher preference for voluntary community-based health insurance (VCHI) compared to OOP payment and suggested that risk aversion on the part of households may have played a role [51]. From this, one can speculate that many households in sub-Saharan Africa prefer the less regressive option of voluntary health insurance to the more regressive and riskier alternative of OOP payments.

Looking broadly at the results one may wonder whether the changes in health financing equity, as observed in this review, reflects progress made in UHC in the countries covered by the review. Available evidence suggests that, at least, in some countries the improvements in health financing equity may have contributed significantly to developments in UHC. However, in others such evidence appears inconclusive. In Thailand, for example, there is a strong indication that achievements in UHC are underpinned by a concerted national effort to strengthen the health financing system to make it more pro-poor and this has led to positive UHC outcomes including equitable access to health services, low level of unmet health needs, and high level of financial risk protection [52]. Annual per capita outpatient visits and inpatient admissions increased from 2.41 and 0.067, respectively, in 2003 to 3.64 and 0.119 in 2011. OP visits among the poorest quintile were disproportionately higher than for the richest quintile. Similarly, IP admissions were concentrated more among the poorest quintile of the population than the wealthiest quintile [52]. In terms of financial risk protection, as a result of the health financing reforms, direct health payments by households fell from 45% of total health spending in 1994 to 15% in 2010 leading to a marked drop in the number of households impoverished by health payment [52]. These achievements coincide with the progress in health financing equity as demonstrated by the BIA and FIA studies in this review.

In the case of sub-Saharan Africa where the majority of the BIA studies show a pro-rich distribution of health benefits, there appears to have been only marginal progress towards UHC. For example, in Kenya, there have been some improvements in access to antenatal care (ANC) in the last decade with deliveries occurring in health facilities increasing from 40% in 2003 to 61% in 2014. However, births by women in the lowest wealth quintile was 16% and 30% in 2003 and 2014 respectively, compared to 74% and 93% by those in the highest quintile for the same period [53,54]. A similar situation can be found in Ghana where deliveries assisted by skilled professionals have increased from 47% in 2003 to 74% in 2014 but assisted deliveries by women from the lowest socioeconomic quintile remained significantly lower (47%) than those for the wealthiest quintile (97%) [55,56]. These, to some extent, confirm the pro-rich distribution of health care benefits as depicted by the BIA studies from the region.

The regressive nature OOP payments in sub-Saharan Africa as evident in the FIA studies also seems to have some practical implications for health seeking behaviour and UHC in the region. Evidence from recent Demographic and Health Surveys (DHS) in many countries demonstrates that most women between the ages of 15–49 face significant financial barriers to accessing health care. In Nigeria, 42% of women cited ‘money’ as a key barrier when it comes to accessing health care. Nearly 53% of women from the lowest wealth quintile compared to 25% of those from the richest quintile made such observation [57]. A similar percentage of women in Ghana (41.7%–59% from the lowest wealth quintile compared to 23% from the richest quintile) and Kenya (36.7%–58% from the lowest wealth quintile compared to 17% from the richest quintile) mentioned ‘money’ as a barrier to accessing health care [54,56]. While there was inadequate data to assess trends over time, these insights corroborate evidence from the FIA studies suggesting that direct payments for health care is more a problem for the poor than the rich.

### Limitations

There were some gaps and inconsistencies in the data presented across the different studies that limited our review. First, in some of the BIA studies the utilisation data used were not disaggregated by type of service (e.g. outpatient–inpatient) and/or by type of facility (hospital, and non-hospital; health centres, clinics, etc.). Without such disaggregation, it is difficult to fully assess the nature of distribution of health care benefits. Second, only a handful of the BIA studies attempted to assess utilisation of private sector health facilities. BIA is typically concerned with the distribution of government resources and hence the assessment of the private sector may seem inconsistent with the dictates of the approach. However, in many LMICs a significant proportion of public funds support private health providers, especially faith-based organisations. For example, the Christian Health Association of Ghana (CHAG) provides an estimated 42% of total health services in the country and about 41% of its operating budget comes from the government of Ghana [58]. Any assessment of health financing equity in LMICs will not be complete without accounting for the utilisation of private sector facilities.

Third, there was no consistency in the way the need for health care was handled across the BIA studies. BIA *per se* does not indicate how much health care a person needs; it only measures how the benefits from public spending on health are distributed across households ranked into socioeconomic groups. It is assumed that people from lower socioeconomic backgrounds generally have higher need for health care than those from wealthier backgrounds. But *how much higher*, *and is the distribution of these benefits in accordance with health care needs*? To address this issue BIA often assesses relative need for health care across households and compares this with the amount of subsidy received. Varied indicators of need are used, usually depending on what datasets are available. For example, many BIA studies in high income countries use *self-assessed health status* to estimate the need for health care across households. Likewise, several national surveys in LMICs include questions on *self-reported illness* which are commonly used to proxy health care need in BIA. There is no consistency in adjusting for or assessing health care need and this may explain some of the difference in benefit incidence across countries.

In addition to these limitations, our method of analysing the data also had some limitations. Several of the BIA studies assessed equity in distribution of health care benefits over time (different years). Although all results were reviewed, we focused largely on the most recent year, which prevented a detailed analysis of changing patterns of health financing equity within countries. However, this review was more interested in equity across LMICs than within countries, and besides, only a few studies undertook such multi-year analysis. The BIA studies conducted by the World Bank evaluated inequality in the incidence of government health spending under three different unit-cost assumptions. In this review we concentrated on the results under the constant unit-cost assumption. Consideration of the other assumptions may have altered the degree of equity in the distribution of benefits reported by those World Bank studies.

Finally, BIA and FIA, as important as they are, are only two of a number of different measures available for assessing the fairness of health care financing. Other measures of equity such as catastrophic health expenditures, which assess the degree of impoverishment of households resulting from health care payments, can be used to measure health financing equity. However, such measures commonly focus on single health financing mechanisms such as OOP spending or social health insurance and do not provide a complete picture of the entire health financing system. As indicated in the methods section, such studies using single measures of equity were excluded from this review because the focus here is on equity of the overall health financing system.

## Conclusions

Equity in health financing in LMICs with regard to the distribution of health care benefits has seen little improvement despite the high priority given to it in recent global initiatives to promote UHC such as the sustainable development goals (SDGs). This systematic review has shown that the distribution of total health care benefits (measured in terms of health service utilisation) benefit the better off more than the poor. Even primary health care services, which are often presumed to be pro-poor, were only marginally so in sub-Saharan Africa. Some countries in Asia-Pacific, namely Thailand, Malaysia and Sri-Lanka, appear to have made significant progress towards distributing health financing in favour of the poor. In terms of the distribution of health financing burden, the vast majority of the studies reviewed found a progressive distribution across all financing sources in Asia-Pacific, but in sub-Saharan Africa, out-of-pocket and voluntary/private health insurance payments were regressive. Overall, policy makers in LMICs, especially those in sub-Saharan Africa, need to increase their efforts to shift government resources towards the poor if the goal of universal health coverage is to be realised.

## Supporting Information

S1 PRISMA Checklist (PDF)
